# Sex steroid metabolism polymorphisms and mammographic density in pre- and early perimenopausal women

**DOI:** 10.1186/bcr2340

**Published:** 2009-07-27

**Authors:** Carolyn J Crandall, Mary E Sehl, Sybil L Crawford, Ellen B Gold, Laurel A Habel, Lesley M Butler, MaryFran R Sowers, Gail A Greendale, Janet S Sinsheimer

**Affiliations:** 1 Department of Internal Medicine, David Geffen School of Medicine, University of California, Los Angeles, UCLA Medicine/GIM, 911 Broxton Ave., 1st floor, Los Angeles, CA 90024, USA; 2 Department of Medicine, David Geffen School of Medicine, University of California, Los Angeles 2333 PVUB Los Angeles, CA 90095-7059, USA; 3 Division of Preventive and Behavioral Medicine, University of Massachusetts Medical School, Worcester, MA, 55 Lake Ave. North, Shaw Building, Worcester, MA 01655, USA; 4 Department of Public Health Sciences, School of Medicine, University of California, Davis, One Shields Ave., TB 168, Davis, CA 95616, USA; 5 Division of Research, Kaiser Permanente, 2000 Broadway, Oakland, CA 94612, USA; 6 Department of Public Health Sciences, University of California at Davis, One Shields Ave., 1616 DaVinci Court, Davis, CA 95616, USA; 7 Department of Epidemiology, School of Public Health, University of Michigan, 109 Observatory, Rm 1846, Ann Arbor, MI 48109-2205, USA; 8 Department of Internal Medicine, David Geffen School of Medicine at University of California, Los Angeles, 10945 Le Conte Ave., Ste. 2339, Los Angeles, CA 90095-1687, USA; 9 Department of Human Genetics, University of California, Los Angeles, 5-357C Gonda/AV268 CHS, Los Angeles, CA 90095-1766, USA

## Abstract

**Introduction:**

We examined the association between mammographic density and single-nucleotide polymorphisms (SNPs) in genes encoding CYP1A1, CYP1B1, aromatase, 17β-HSD, ESR1, and ESR2 in pre- and early perimenopausal white, African-American, Chinese, and Japanese women.

**Methods:**

The Study of Women's Health Across the Nation is a longitudinal community-based cohort study. We analyzed data from 451 pre- and early perimenopausal participants of the ancillary SWAN Mammographic Density study for whom we had complete information regarding mammographic density, genotypes, and covariates. With multivariate linear regression, we examined the relation between percentage mammographic breast density (outcome) and each SNP (primary predictor), adjusting for age, race/ethnicity, parity, cigarette smoking, and body mass index (BMI).

**Results:**

After multivariate adjustment, the *CYP1B1 *rs162555 CC genotype was associated with a 9.4% higher mammographic density than the TC/TT genotype (*P *= 0.04). The *CYP19A1 *rs936306 TT genotype was associated with 6.2% lower mammographic density than the TC/CC genotype (*P *= 0.02). The positive association between *CYP1A1 *rs2606345 and mammographic density was significantly stronger among participants with BMI greater than 30 kg/m^2 ^than among those with BMI less than 25 kg/m^2 ^(P_interaction _= 0.05). Among white participants, the *ESR1 *rs2234693 CC genotype was associated with a 7.0% higher mammographic density than the CT/TT genotype (*P *= 0.01).

**Conclusions:**

SNPs in certain genes encoding sex steroid metabolism enzymes and ESRs were associated with mammographic density. Because the encoded enzymes and ESR1 are expressed in breast tissue, these SNPs may influence breast cancer risk by altering mammographic density.

## Introduction

High mammographic breast density, the density of the breast on mammography, is one of the strongest known risk factors for breast cancer [1]. High breast density (dense tissue on 50% or more of the breast) could account for up to one third of breast cancer cases [2]. Factors such as body mass index, parity, age, smoking, and physical activity jointly account for only a small proportion of the variability in mammographic density [3]. In contrast, mammographic density has a strong genetic component. Twin studies have demonstrated that heritability (the proportion of variance attributable to genetic factors) accounts for 60% of the variance in mammographic density [4,5].

It is feasible that genetic variation in sex steroids or in estrogen receptors (ESRs) produced in breast tissue could lead to differing degrees of proliferation that may be manifest radiographically as interindividual differences in mammographic density. The presence of sex steroid metabolic enzymes and ESRs in breast tissue [6-24] suggests that local activation of estrogen to potentially reactive metabolites within breast tissue may play a role in initiating and promoting carcinogenesis [18]. Such enzymes include CYP1A1, CYP1B1, and 17β-hydroxysteroid dehydrogenase (17β-HSD). In addition to metabolizing environmental carcinogens (for example, polycyclic aromatic hydrocarbons), CYP1A1 has high activity with the 17β-estradiol substrate [25,26]. CYP1A1 forms mainly 2-hydroxyestrone, and to a lesser degree, some 4-hydroxyestrone, from estrone. In contrast, CYP1B1 predominantly catalyzes formation of potentially carcinogenic catechol estrogens, especially 4-hydroxyestrogens [6,26-28]. The implication of 4-hydroxy catechol estrogens in carcinogenesis suggests a key role for CYP1B1 in carcinogenesis [19,27,29,30]. *CYP19A1 *is the gene encoding the aromatase enzyme that catalyzes the formation of aromatic C18 estrogens from C19 androgens [6,31]. Type I 17β-HSD is the enzyme responsible for interconversion of estrone and estradiol [32]. In addition to potential local effects of these enzymes on breast tissue, ESR-estrogen interactions stimulate breast epithelial cell growth [33]. Single-nucleotide polymorphisms (SNPs) in genes encoding sex steroid-metabolizing enzymes or receptors have effects on the hormonal milieu of the breast and on levels of potential mammary carcinogens [6].

A few studies have explored associations between mammographic density and SNPs in genes encoding CYP1A1, CYP1B1, aromatase, 17β-HSD, ESR1, and ESR2 [34-39]. However, most studies were focused on postmenopausal women [36,37]. Premenopausal breast density may be more highly heritable than is postmenopausal density [40], and some genes may be associated with premenopausal but not with postmenopausal density [4]. The goal of this study was to examine the association between mammographic density and SNPs in genes encoding CYP1A1, CYP1B1, aromatase, type I 17β-HSD, ESR1, and ESR2 in a group of pre- and early perimenopausal white, African-American, Chinese, and Japanese women.

## Materials and methods

To determine the association between SNPs in genes encoding sex steroid-metabolizing enzymes and ESRs and mammographic density, we used data from women who participated in the SWAN ancillary Mammographic Density Study and the SWAN Genetics Study, which are described later. All protocols were IRB approved at participating sites, and all participating women provided signed, written informed consent.

### The Study of Women's Health Across the Nation (SWAN)

SWAN is a multisite longitudinal community-based cohort study of 3,302 midlife women, serving as the parent study for the Mammographic Density ancillary study. In brief, at baseline, women were aged 42 to 52 years and premenopausal (reporting no change in usual menstrual pattern) or early perimenopausal (reporting change in menstrual pattern but occurrence of menstruation in the past 3 months), had an intact uterus and one or more ovaries, were not pregnant or lactating, and were not using exogenous reproductive hormones [41]. Initiation of exogenous hormones after the baseline visit did not preclude inclusion in the longitudinal cohort study. Each of the seven study sites enrolled white women in addition to women of one other self-identified racial/ethnic group: African-American women (Boston, Detroit area, Chicago, and Pittsburgh), Japanese women (Los Angeles), Hispanic women (New Jersey), and Chinese women (Oakland, California). SWAN participants completed questionnaires and underwent fasting blood sampling annually.

### The SWAN Mammographic Density Study

Three SWAN clinical sites (Los Angeles, Oakland, Pittsburgh) participated in the SWAN Mammographic Density ancillary study, which retrieved and analyzed existing participants' mammograms that had been performed by accredited mammography facilities as a part of routine medical care.

At the time of enrollment into the ancillary study, 1,248 participants were active at the three sites. Of these, 22 (2%) women were ineligible because of bilateral breast surgery, 82 (7%) were not recruited because of having an abbreviated follow-up, and 89 (7%) refused to participate. Thus, 1,055 (85%) women were eligible and agreed to participate in the mammographic density study; of these, 1,005 women had at least one mammographic density assessment.

By using previously published methods, a single expert reviewer quantified mammographic density (that is, the percentage of the breast composed of dense tissue) [42]. The reader assessed mammographic density by using the craniocaudal view of the mammogram of the right breast [43]. If a participant reported prior breast surgery involving the right breast, mammograms of the left breast were used for density assessments. A compensating polar planimeter was used to measure the total breast area (in square centimeters) and the area of dense breast tissue (in square centimeters). Percentage density was calculated as the area of dense breast tissue divided by the area of the breast. A repeated review of a 10% random subset of mammograms for intrarater reliability yielded an intraclass correlation coefficient for percentage mammographic density of 0.96 [43].

Our goal was to examine associations of SNPs with mammographic density among pre- and early perimenopausal participants. Of the 1,005 participants with at least one assessment of mammographic density, we chose one mammogram for each of the 643 pre- or early perimenopausal SWAN Mammographic Density study participants. If more than one mammogram was available for given participant, we selected the mammogram temporally closest to the preceding annual follow-up visit that was flanked by pre- or early perimenopausal status on the visits before and after the mammogram. For example, if a participant had mammographic density assessments from two mammograms during her premenopausal stage and one mammogram during her early perimenopausal stage, we chose a single mammogram for the participant by picking the mammogram that was temporally closest to its preceding annual follow-up visit. Mammograms that occurred more than 3 months before baseline and mammograms obtained during the use of current exogenous reproductive hormones were excluded.

### The SWAN Genetics Study

**Figure 1:**
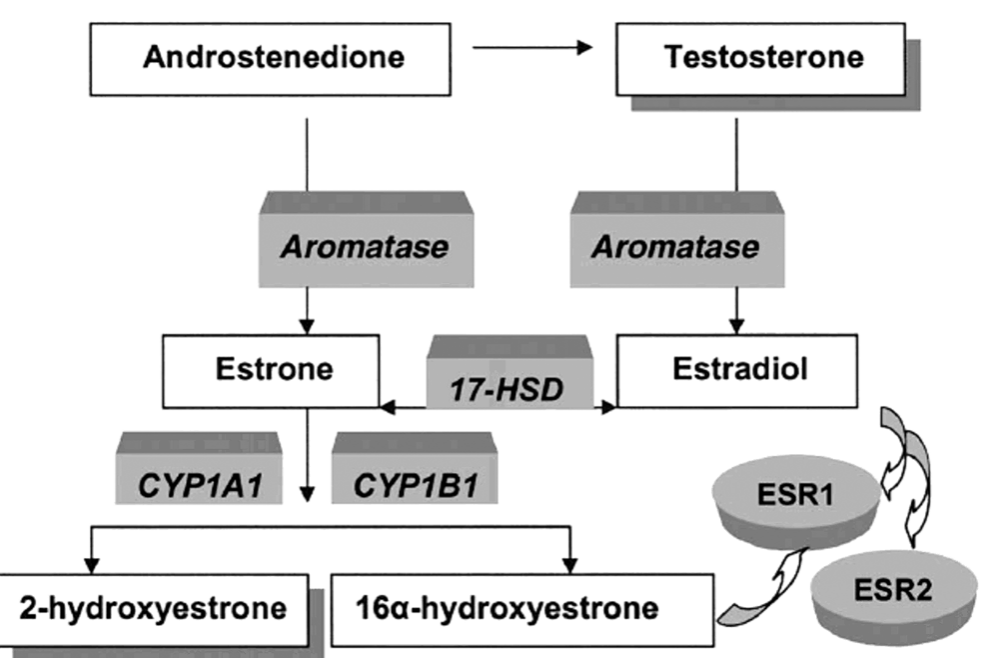
Functions of SWAN genetics sex steroid metabolism enzymes and receptors. Used with permission of Sowers and colleagues [93].

The SWAN Genetics Study genotyped 25 SNPs relating to sex-steroid metabolism and estrogen receptors (Figure 1, Table 1). Of the 1,988 women who were eligible (that is, still participating and providing blood for the SWAN parent study at the follow-up year 5 visit), 88% agreed to participate in the genetics study. Details regarding specimen collection, specimen processing, and genotyping were previously reported [44]. Genotyping was performed by using TaqMan (Roche Molecular Systems, Inc., Pleasanton, CA) and an ABI 7900 HT sequence detection system (Applied Biosystems Inc., Foster City, CA, USA).

**Table 1:** SNPs examined in the SWAN Genetics Study

rs Number	Other common designation of SNP	Affected alleles^a^	Region of affected DNA sequence	Change in amino acid
**17-β HSD chromosome 17q11-q21**				
rs615942	HSD615942^b^	G/T	Missense, amino acid position 55, codon position 2, C → A exon 2	Ser → Tyr
rs592389	HSD592389	G/T	3' Near gene	N/A
rs2830	HSD17B2830	A/G	5' Untranslated region exon 1	N/A
**ESR1 chromosome 6q25.1**				
rs9340799	ESRA464, *Xba*I RFLP	A/G	Intron 1	N/A
rs2234693	ESRA418, *Pvu*II RFLP	T/C	Intron 1	N/A
rs728524	ESR728524	A/G	Intron 4	N/A
rs3798577	ESR3798577	T/C	3' Untranslated region exon 8	N/A
**ESR2 chromosome 14q23.2**				
rs1256030	ESR1256030	C/T	Intron 2	N/A
rs1255998	ESR1255998	C/G	3' Untranslated region exon 9	N/A
rs1256049	ESR1256049, V328V, *Rsa*I	G/A	Synonymous, amino acid position 328 (valine), codon position 3 exon 6	N/A
**CYP1A1 chromosome 15q22-q24**				
rs2606345	CYP2606345, -1806	C/A	Intron 1	N/A
rs4646903	CYP1A1250, *Msp*I, m1, T6235C	T/C	3' Near gene	N/A
rs1531163	CYP1531163, -11781 promoter^c^	A/G	Synonymous, amino acid position 294 (lysine), codon position 3 exon 8	N/A
rs1048943	CYP1048943, CYP1A1*2C, A2455G, m2, 6750 A>G	A/G	Missense, amino acid position 462, codon position 1 A → G exon 7	Ile → Val
**CYP1B1 chromosome 2p21**				
rs1800440	CYP1800440, N453S, A4390G, CYP1B1*4	A/G	Missense, amino acid position 453, codon position 2 A → G exon 3	Asn → Ser
rs162555	CYP162555 ^d^	T/C	Intron 2	N/A
rs1056836	CYP1056836, CYP1B1*3, Leu432Val, 4326C>G, C1294G, m1	C/G	Missense, amino acid position 432, codon position 1 G → C exon 3	Val → Leu
**CYP19A1 chromosome 15q21.1**				
rs700519	CYP19R264	C/T	Missense, amino acid position 264, codon position 1 C → T exon 7	Arg → Cys
rs2414096	CYP194096	G/A	Intron 2	N/A
N/A	CYP194947, hCV8234946	A/G	Intron 1	N/A
rs1008805	CYP198805	T/C	Intron 1	N/A
rs2446405	CYP196405^a^	T/A	Intron 1	N/A
rs2445759	CYP195759	G/T	Intron 1	N/A
rs936306	CYP196306	C/T	Intron 1	N/A
rs749292	CYP199292	G/A	Intron 1	N/A

^a^Reference (higher frequency) allele is listed first. ^b^Encodes Coenzyme A synthase. ^c^Encodes lectin, mannose-binding, 1-like. ^d^Encodes LOC732 hypothetical protein. ^e^Encodes gliomedine.

Between three and eight SNPs per gene were selected based on use in previous genetics studies, a review of the literature, and information from gene databases (National Center for Biotechnology SNP database [45] and Celera [46]). The original SNP selection process is discussed in the first SWAN Genetics Study manuscript [44]. The SWAN Genetics Study searched for published literature supporting the biologic significance of SNPs chosen. SNPs were chosen if they were thought potentially to influence circulating sex hormone levels [47,48] or disease patterns: breast cancer [49-51], ovarian cancer [52], and bone mineral density [53,54].

Of the 643 premenopausal or early perimenopausal SWAN participants with available mammographic density information, at least partial genotyping data were available for 463 (72%) women. For this analysis, we excluded one participant lacking genotyping data for 24 of the 25 SNPs and an additional 11 participants who were missing information for one or more covariates. Thus, the analytic sample for this study comprised the 451 pre- and early perimenopausal women for whom complete information was available regarding mammographic density, genotypes, and covariates.

### Questionnaire-based and anthropometric measures

At baseline and at each annual follow-up visit, SWAN participants were asked to complete standardized questionnaires and underwent measurement of height and weight for calculation of body mass index (BMI, weight in kilograms divided by the square of the height in meters). We took information regarding age, race/ethnicity, reproductive history, medication use, smoking, and alcohol intake from annual questionnaires.

### Statistical analysis

Allele frequencies in the SWAN Genetics Study were estimated by race/ethnicity (Mendel Version 8.0 [55]). Hardy-Weinberg equilibrium (HWE) was assessed by using Fisher's Exact tests [55]. Because of the multiple statistical tests performed, we considered a *P *value of < 0.01 as the criterion to reject the null hypothesis of HWE.

Creating a separate model for each of the 25 SNPs, we used multivariate linear regression to examine the relation between percentage mammographic breast density (outcome) and SNP (primary predictor). Based on previously published studies, we considered the following candidate covariates: age, race/ethnicity, number of live births, BMI, oral contraceptive use, menopausal hormone use, cigarette smoking, and alcohol intake) [3,43,56-68]. Of these candidate covariates, age, number of live births, and BMI were included in all models, based on previously well-established associations with mammographic density. The remaining candidate covariates (cigarette smoking, alcohol intake, oral contraceptive use, and menopausal hormone use) were evaluated for model inclusion by using backwards regression performed on data from the 643 pre- and early perimenopausal participants of the SWAN Mammographic Density study. We used a *P *value of 0.10 as the cutoff for covariate inclusion. In addition, because each site recruited a specific racial/ethnic group in addition to non-Hispanic whites, a combined variable was created for race/ethnicity and study site; this variable was included in all models. Categories for this variable were whites in Oakland, Chinese in Oakland, whites in Los Angeles, Japanese in Los Angeles, white in Pittsburgh, or African American in Pittsburgh. Age at mammogram (continuous), race/ethnicity-study site, number of live births (continuous), current cigarette smoking (yes/no), and BMI (continuous) were the covariates retained in the final models. We modeled the alleles as acting in either an additive (aa versus Aa and AA, where the effect of the Aa genotype is half the effect of the AA genotype) or recessive (aa/Aa versus AA) manner, in which A is the minor allele.

Because of prior studies showing that associations of sex steroid-related SNPs may be more evident among women with BMI greater than 25 [69], we conducted secondary analyses wherein we added an SNP*BMI interaction term to the multivariable linear regression models. Because we suspected that the sample size of certain racial/ethnic subgroups may have been too small to allow detection of SNP*race/ethnicity interactions, and because the allele frequencies for 13 of the 25 SNPs differed by more than 0.20 between the ethnic groups, we repeated all of our analyses in the subsample of white participants, the largest racial/ethnic subgroup. All regression analyses were performed with the software program R [70].

## Results

### Baseline characteristics of the participants

Baseline characteristics of the analytic sample (N = 451 with mammographic density, genotyping, and covariate data) are displayed in Table 2. No notable differences in characteristics were found between the overall mammographic density sample (N = 643) and the analytic sample. The median age of the participants in the analytic sample was 48.7 years (Table 2). Median BMI was 24.4 kg/m^2^. Mean percentage mammographic density was 43.6%. Forty-nine percent of the participants in the analytic sample were white, 24% were Chinese, 22% were Japanese, and 6% were African American. Twenty-six percent were premenopausal, and 74% were early perimenopausal at the visit immediately preceding mammography (Table 2).

**Table 2:** Characteristics of the study participants: analytic sample of the current study (N = 451)

	Mean	SD	Median	Number	%
Age at mammogram, years	48.6	2.6	48.7		
BMI, kg/m^2^	25.9	5.9	24.4		
<25				251	56
25–30				111	25
>30				89	20
Percentage mammographic density	43.6	19.5	44.7		
Age at first full-term birth	28.1	5.5	28.0		
Ethnicity					
African American				26	6
White				219	49
Chinese				109	24
Japanese				97	22
Study site					
Oakland				184	41
Los Angeles				169	37
Pittsburgh				98	22
Smoking currently				31	7
Menopausal status at time of mammogram					
Early perimenopausal				332	74
Premenopausal				118	26
Parity					
0				77	17
1				76	17
2				196	43
3				76	17
4				19	4
≥ 5				7	2

Baseline menopause stage information was missing for one participant whose baseline mammogram was chosen; the participant was early perimenopausal at the subsequent (first annual) visit.

### Hardy-Weinberg Equilibrium assessment

We examined allele frequencies by ethnicity (Table 3) and assessed HWE (see Table 4). Within racial/ethnic subgroups, only *CYP194947 *showed significant deviation from HWE. Because this SNP was also the SNP with the highest frequency (8%) of missing genotypes, methodologic considerations related to genotyping of this SNP may have contributed to deviation from HWE.

**Table 3:** Allele frequencies of SWAN Genetics Study participants by race/ethnicity

		Estimated frequencies				
Locus	Allele	All	African	White	Chinese	Japanese

**17β-HSD**						
rs615942	G	0.52	0.64	0.47	0.55	0.56
	T	0.48	0.36	0.53	0.45	0.44
rs592389	A	0.50	0.41	0.475	0.56	0.56
	C	0.50	0.59	0.53	0.44	0.44
rs2830	A	0.50	0.59	0.53	0.44	0.44
	G	0.50	0.41	0.47	0.56	0.56
**ESR1**						
rs9340799	A	0.72	0.73	0.65	0.77	0.82
	G	0.28	0.27	0.35	0.23	0.18
rs2234693	C	0.46	0.46	0.46	0.41	0.48
	T	0.54	0.54	0.54	0.59	0.52
rs728524	A	0.89	0.76	0.98	0.81	0.81
	G	0.11	0.24	0.02	0.19	0.188
rs3798577	C	0.46	0.41	0.48	0.45	0.45
	T	0.54	0.59	0.52	0.55	0.55
**ESR2**						
rs1256030	C	0.61	0.75	0.57	0.70	0.58
	T	0.39	0.25	0.43	0.30	0.42
rs1255998	C	0.68	0.50	0.89	0.37	0.54
	G	0.32	0.50	0.11	0.63	0.46
rs1256049	A	0.18	0.11	0.02	0.45	0.30
	G	0.82	0.89	0.98	0.55	0.70
**CYP1A1**						
rs2606345	A	0.37	0.18	0.66	0.06	0.05
	C	0.63	0.82	0.34	0.94	0.95
rs4646903	A	0.75	0.71	0.89	0.54	0.61
	G	0.25	0.29	0.11	0.46	0.39
rs1531163	A	0.88	0.66	0.95	0.84	0.80
	G	0.12	0.34	0.05	0.16	0.20
rs1048943	A	0.88	0.98	0.96	0.75	0.76
	G	0.12	0.02	0.04	0.25	0.24
**CYP1B1**						
rs1800440	A	0.89	0.90	0.80	1.00	1.00
	G	0.11	0.10	0.20	0.004	0.006
rs162555	C	0.12	0.16	0.19	0.04	0.01
	T	0.88	0.84	0.81	0.96	0.99
rs1056836	C	0.69	0.33	0.59	0.88	0.86
	G	0.31	0.67	0.41	0.12	0.14
**CYP19**						
rs700519	A	0.12	0.13	0.04	0.14	0.29
	G	0.88	0.88	0.96	0.86	0.71
rs2414096	A	0.43	0.25	0.50	0.46	0.32
	G	0.57	0.75	0.50	0.54	0.68
4947^a^	A	0.52	0.71	0.55	0.39	0.49
	G	0.48	0.29	0.45	0.61	0.51
rs1008805	C	0.36	0.16	0.42	0.28	0.37
	T	0.64	0.84	0.58	0.72	0.63
rs2446405	A	0.32	0.52	0.18	0.47	0.45
	T	0.68	0.48	0.82	0.53	0.55
rs936306	C	0.73	0.40	0.85	0.70	0.60
	T	0.27	0.60	0.15	0.30	0.40
rs2445759	G	0.96	0.92	0.93	1.00	1.00
	T	0.04	0.08	0.07	0.004	0.003
rs749292	A	0.44	0.51	0.44	0.50	0.36
	G	0.56	0.49	0.56	0.50	0.64

^a^No corresponding dbsnp rs number; commonly referred to as CYP194947.

**Table 4:** Hardy-Weinberg equilibrium evaluation by race/ethnicity

	All		African		White		Chinese		Japanese	
	
Locus	*P *value	Number	*P *value	Number	*P *value	Number	*P *value	Number	*P *value	Number
HSD										

**rs615942**	**0.0741**	**716**	**1**	**56**	**0.0691**	**364**	**0.2962**	**139**	**0.632**	**157**
**rs592389**	**0.0462**	**714**	**0.4159**	**56**	**0.075**	**361**	**0.3061**	**138**	**0.7462**	**156**
**rs2830**	**0.0356**	**715**	**0.4074**	**56**	**0.1147**	**362**	**0.1189**	**139**	**0.7504**	**156**
ESR1										
**rs9340799**	**0.5221**	**720**	**0.1818**	**56**	**0.1726**	**365**	**0.6308**	**140**	**0.5958**	**157**
**rs2234693**	**0.8786**	**718**	**0.5964**	**56**	**0.5327**	**364**	**1**	**139**	**0.4255**	**157**
**rs728524**	**0.0724**	**719**	**0.4825**	**56**	**1**	**364**	**1**	**140**	**0.7874**	**157**
**rs3798577**	**0.9362**	**715**	**0.7783**	**55**	**0.8299**	**363**	**1**	**139**	**1**	**156**
ESR2										
**rs1256030**	**0.6411**	**715**	**0.0811**	**56**	**1**	**363**	**0.1591**	**140**	**0.7379**	**154**
**rs1255998**	<0.00005	**712**	**0.2985**	**56**	**0.41**	**361**	**0.199**	**138**	**1**	**155**
**rs1256049**	<0.00005	**679**	**1**	**46**	**0.1804**	**344**	**0.3854**	**132**	**0.5638**	**155**
CYP1A1										
**rs2606345**	<0.00005	**715**	**0.6693**	**55**	**0.9088**	**360**	**1**	**141**	**1**	**157**
**rs4646903**	<0.00005	**706**	**0.7502**	**55**	**0.4137**	**356**	**1**	**138**	**0.1252**	**155**
**rs1531163**	0.0092	**712**	**0.5642**	**56**	**0.5508**	**359**	**0.3384**	**139**	**0.3173**	**156**
**rs1048943**	0.0001	**714**	**1**	**56**	**1**	**362**	**0.6582**	**138**	**0.0452**	**156**
CYP1B1										
**rs1800440**	<0.00005	**685**	**0.347**	**47**	**0.0629**	**345**	**1**	**135**	**1**	**156**
**rs162555**	0.0033	**716**	**1**	**56**	**0.2346**	**364**	**0.1518**	**138**	**1**	**156**
**rs1056836**	<0.00005	**704**	**0.0591**	**53**	**0.2778**	**355**	**1**	**139**	**0.3159**	**155**
CYP19										
**rs700519**	**0.0756**	**712**	**0.1854**	**56**	**1**	**360**	**0.0694**	**138**	**0.7032**	**156**
**rs2414096**	**0.9384**	**719**	**0.7184**	**56**	**0.1193**	**365**	**0.2394**	**141**	**0.3603**	**155**
**4947^a^^¶^**	<0.00005	654	0.0195	41	0.5857	344	<0.00005	129	<0.00005	138
**rs1008805**	**0.7465**	**717**	**0.6143**	**56**	**0.2444**	**364**	**0.2942**	**140**	**0.7366**	**155**
**rs2446405**	**0.0376**	**716**	**0.7845**	**55**	**0.7187**	**364**	**0.6048**	**139**	**0.7456**	**156**
**rs936306**	0.0031	**717**	**1**	**56**	**0.3074**	**364**	**0.552**	**140**	**0.7366**	**155**
**rs2445759**	**1**	**682**	**1**	**45**	**1**	**344**	**1**	**135**	**1**	**156**
**rs749292**	**0.6463**	**715**	**0.4183**	**55**	**0.2371**	**361**	**0.6075**	**141**	**1**	**156**

^a^No corresponding dbsnp rs number; commonly referred to as CYP194947.

### Associations between SNPs and percentage mammographic density

We examined the association between percentage mammographic breast density and each of the SNPs in models adjusted for age, race/ethnicity-study site, parity, smoking, and BMI (Tables 5 and 6).

**Table 5:** Percentage mammographic density as a function of single-nucleotide polymorphism: recessive models^a^

	Entire analytic sample (N = 451)			Whites only (N = 219)		
	Age, race/ethnicity-study site,^b ^parity, smoking, body mass index			Age, study site, parity, smoking, body mass index		

Locus	β	SD	*P *value	β	SD	*P *value

**17-β HSD**						
rs615942 G	-0.18	1.73	0.92	0.78	2.55	0.76
rs592389 A	-0.81	1.71	0.64	0.97	2.55	0.70
rs2830 A	1.75	1.65	0.29	2.69	2.59	0.30
**ESR1**						
rs9340799 A	4.40	2.79	0.12	6.67	3.50	0.06
rs2234693 T	3.37	1.81	0.06	7.04	2.67	**0.01**
rs728524 A	1.43	5.33	0.79	N/A	N/A	N/A
rs3798577 T	3.04	1.79	0.09	1.14	2.75	0.68
**ESR2**						
rs1256030 C	0.72	2.10	0.73	0.80	2.94	0.79
rs1255998 C	-0.14	2.25	0.95	-11.59	11.92	0.33
rs1256049 G	-5.58	3.21	0.08	-6.98	16.85	0.68
**CYP1A1**						
rs2606345 C	-0.21	2.15	0.92	-0.09	2.32	0.97
rs4646903 A	0.83	2.45	0.73	-1.11	6.45	0.99
rs1531163 A	0.09	4.47	0.98	-5.50	16.87	0.74
rs1048943 A	1.14	3.99	0.77	N/A	N/A	N/A
**CYP1B1**						
rs1800440 A	-2.29	3.92	0.56	-1.35	4.50	0.76
rs162555 T	9.36	4.62	**0.04**	8.23	5.45	0.13
rs1056836 C	2.48	2.51	0.33	1.37	3.07	0.66
**CYP19A1**						
rs700519 G	-4.59	6.12	0.45	N/A	N/A	N/A
rs2414096 G	-0.83	1.94	0.67	1.33	2.81	0.64
CYP194947^¶ ^A	1.30	1.68	0.44	3.93	2.83	0.17
rs1008805 T	-3.56	2.24	0.11	-2.36	3.07	0.44
rs2446405 T	1.99	2.31	0.39	-4.96	5.78	0.39
rs936306 C	-6.17	2.82	**0.03**	-16.36	5.96	**0.01**
rs2445759 G	-7.30	9.18	0.43	-10.36	11.98	0.39
rs749292 G	0.40	1.93	0.84	3.53	2.98	0.24

^a^Reference allele was the allele with the higher frequency in the overall analytic sample and is indicated in the first column for each SNP.

^b^Because each site recruited a specific racial/ethnic group in addition to non-Hispanic whites, a combined variable was created for race/ethnicity and study site.

**Table 6:** Percentage mammographic density as a function of single-nucleotide polymorphism: additive models^a^

	Entire analytic sample (N = 451)			Whites only (N = 219)		
	Age, race/ethnicity-study site^b^, parity, smoking, body mass index			Age, study site, parity, smoking, body mass index		

						

LOCUS	β	SD	*P *value	β	SD	*P *value

**17-β HSD**						
rs615942 G	-0.68	1.02	0.50	-0.68	1.55	0.66
rs592389 G	-1.01	1.02	0.32	-0.59	1.56	0.71
rs2830 A	1.05	1.01	0.30	0.63	1.56	0.69
**ESR1**						
rs9340799 A	1.58	1.22	0.20	3.11	1.77	0.08
rs2234693 T	1.44	1.06	0.18	4.08	1.61	**0.01**
rs728524 A	3.35	1.77	0.06	-9.14	6.50	0.16
rs3798577 T	1.01	1.05	0.34	-0.36	1.65	0.83
**ESR2**						
rs1256030 C	0.79	1.10	0.47	0.76	1.64	0.64
rs1255998 C	-0.42	1.30	0.75	-2.38	2.61	0.36
rs1256049 G	-1.02	1.53	0.51	-2.28	4.34	0.60
**CYP1A1**						
rs2606345 C	-1.32	1.40	0.34	-1.00	1.69	0.56
rs4646903 T	1.49	1.19	0.21	1.34	2.25	0.55
rs1531163 A	-0.33	1.60	0.84	-4.51	3.70	0.23
rs1048943 A	0.20	1.55	0.90	1.19	4.05	0.77
**CYP1B1**						
rs1800440 A	0.72	1.58	0.65	1.73	1.84	0.35
rs162555 T	0.52	1.64	0.75	0.45	2.01	0.82
rs1056836 C	-0.37	1.22	0.78	-1.39	1.62	0.39
**CYP19A1**						
rs700519 C	1.59	1.72	0.36	-3.46	4.00	0.39
rs2414096 G	0.77	1.11	0.49	2.75	1.74	0.12
CYP194947^c ^A	0.99	0.96	0.30	2.67	1.62	0.10
rs1008805 T	-0.02	1.12	0.99	-0.65	1.66	0.70
rs2446405 T	0.81	1.20	0.50	-3.02	2.05	0.14
rs936306 C	-0.92	1.25	0.46	-3.67	2.12	0.09
rs2445759 G	-3.47	2.36	0.14	-5.19	2.86	0.07
rs749292 G	0.67	1.08	0.53	2.83	1.67	0.09

^a^Reference allele was the allele with the higher frequency in the overall analytic sample and is indicated in the first column for each SNP. ^b^Because each site recruited a specific racial/ethnic group in addition to non-Hispanic whites, a combined variable was created for race/ethnicity and study site. ^c^No corresponding dbsnp rs number; commonly referred to as CYP194947.

In the fully adjusted recessive models (adjusted for age, race/ethnicity-study site, parity, smoking, and BMI), the *CYP1B1 *rs162555 CC genotype was associated with 9.4% higher percentage mammographic density than the TC/TT genotype (*P *= 0.04). The *CYP19A1 *rs936306 TT genotype was associated with 6.2% lower percentage mammographic density than TC/CC genotype (*P *= 0.03) (Table 5). In contrast to analyses restricted to white participants, *ESR1 *rs2234693 was not significantly associated with mammographic density in either recessive or additive models that included the entire analytic sample (Tables 5 and 6).

### Interaction by BMI

In additive models, *CYP1A1 *rs2606345 was significantly associated with BMI (1.1 kg/m^2 ^higher for each A allele; *P *= 0.03) and *CYP19A1 *rs2414096 (1.1 kg/m^2 ^lower for each A allele; *P *= 0.01; data not shown). Similarly, in recessive models restricted to white participants, the *CYP194947 *GG genotype was associated with a 2.1 kg/m^2 ^lower BMI compared with the GA/AA genotype (*P *= 0.05), and the *CYP19A1 *rs749292 AA genotype was associated with a 2.3 kg/m^2 ^lower BMI than the GA/GG genotype (*P *= 0.05; data not shown).

To determine whether associations between SNPs and mammographic density varied according to BMI, we added BMI*SNP interaction terms to multiple linear regression models that included age, race/ethnicity-study site, smoking, parity, and BMI as covariates and percentage mammographic density as the outcome (data not shown). In additive models, the *CYP1A1 *rs2606345-mammographic density association was significantly different (stronger) among participants with BMIs greater than 30 kg/m^2 ^compared with participants with BMIs less than 25 kg/m^2 ^(P_interaction _= 0.05). Specifically, among participants with BMIs less than 25 kg/m^2^, percentage mammographic density was 0.57% higher for each *CYP1A1 *rs2606345 C allele; in contrast, among participants with BMIs greater than 30 kg/m^2^, percentage mammographic density was 6.1% higher for each additional C allele. The associations of SNPs with mammographic density did not significantly differ by BMI category for *CYP19A1 *rs2414096, *CYP19A1 *rs749292, or *CYP194947*. However, we may not have had adequate statistical power to detect an SNP*BMI interaction when BMI was categorized into tertiles.

### Analyses restricted to white participants

In analyses restricted to whites (n = 219), we detected two associations that were similar to those seen in the overall analytic sample (for example, *CYP19A1 rs936306 *in recessive models, *CYP19A1 *rs2414096 in additive models). In white participants, the *ESR1 *rs2234693 CC genotype was associated with a 7.0% higher percentage mammographic density than the CT/TT genotype (*P *= 0.01; Table 5); this finding was also apparent in additive models (*P *= 0.01; Table 6). The association between *ESR1 *rs2234693 and mammographic density varied by ethnicity; the association was stronger among whites than among Japanese (interaction *P *value, 0.09) or Chinese (interaction *P *value, 0.03) participants.

## Discussion

In pre- and early perimenopausal women, SNPs involving *CYP1B1 *(rs162555 CC genotype), *CYP19A1 *(rs936306 TT/CC genotype), and *ESR1 *(rs2234693 CC genotype) were each significantly positively associated with mammographic density. Associations between several SNPs (*CYP1A1 *rs2606345, *CYP194947*, *CYP19A1 *rs749292, *CYP19A1 *rs2414096) and mammographic density were attenuated after adjustment for BMI. Percentage mammographic density varied at least 3% per allele for the statistically significant associations. These differences in mammographic density according to genotype are of a clinically relevant magnitude, given that each 1% increment in mammographic density is associated with a 2% higher relative risk of breast cancer [2]. Several SNP-mammographic density associations varied significantly by ethnicity.

Several of our findings are novel. As far as we know, other publications have not reported information regarding associations between mammographic density and the following SNPs: *17β-HSD *rs615942, *17β-HSD *rs592389, *17β-HSD *rs2830, *ESR1 *rs728524, *ESR1 *rs3798577, *ESR2 *rs1256030, *ESR2 *rs1255998, *ESR2 *rs1256049, *CYP1B1 *rs162555, *CYP1B1 *rs1800440, *CYP19A1 *rs700519, *CYP19A1 *rs2446405, *CYP19A1 *rs2445759, *CYP19A1 *rs1008805, *CYP19A1 *rs936306, *CYP19A1 *rs2414096, *CYP19A1 *rs749292, *CYP194947*, *CYP1A1 *rs1531163, or *CYP1A1 *rs2606345.

Our finding of an association between *ESR1 *rs2234693 and mammographic density among white women conflicts with some prior studies. The association between *ESR1 *rs2234693 and mammographic density was described in three reports from the EPIC study. In the first EPIC report, the T allele was associated with higher mammographic density [39], whereas in this study, the CC genotype is associated with higher mammographic density.

The second EPIC analysis found a statistically significant difference in mammographic density between hormone therapy users and never-users of hormone therapy among women the CT or TT genotype, but not among those with the CC genotype [71].

The third EPIC analysis reported no association between *ESR1 *rs2234693 and mammographic density [36]; the discrepancy among studies may be because the previous study used a different mammographic density measurement technique, had a less heterogenous study population, and focused on postmenopausal women.

We found an association between *CYP1B1 *rs1056836 and mammographic density that neared statistical significance only before adjustment, but not after adjustment, for BMI. These results may be consistent with three previously published studies [35,36,38].

A cross-sectional observational European study of white women found statistically significantly higher mammographic density in carriers of at least one *ESR1 *rs9340799 A allele [39]. Although we had similar results, our findings were not statistically significant, possibly because of the smaller number of participants in our study or the younger age of our participants.

The other SNPs involved in sex steroid metabolism or estrogen receptors were not significantly associated with mammographic density in the present study. As with our study, past studies reported absence of an association between mammographic density and *CYP1A1 *rs1048943 and *CYP1A1 *rs4646903 [35,38].

Although previously published studies have not included a systematic examination of sex steroid metabolism SNPs and mammographic density, some previously studied SNPs may be linked with the SNPs that we examined. We searched Haploview version 4.1 (Daly Lab, Cambridge, MA) with Hapmap genotype data to search for information regarding linkage disequilibrium for each of the three SNPs that we found to be associated with mammographic density and other SNPs previously studied in relation to mammographic density. Linkage diseqilibrium *R*^2 ^values for *ESR1A1 *rs2234693 (which we found to be associated with mammographic density) and rs9340799 (which prior studies found to be associated with mammographic density) range from 0.234 to 0.55, depending on the ethnic group. For *CYP19A1 *rs936306 (which we found to be associated with mammographic density) and rs10046 (which prior studies found not to be associated with mammographic density), *R*^2 ^values range from 0.017 to 0.193. Linkage-disequilibrium information is not currently available for *CYP1B1 *rs162555 on Hapmap. Although LD information was not available for rs162555, we note that its chromosomal location is not close to the other two previously studied *CYP1B1 *SNPs.

Our findings have a biologic rationale. A local influence of sex steroid metabolism SNPs on breast tissue is suggested by prior breast cancer studies. For example, *ESR1 *rs2234693 has been associated with duration of breast cancer survival [72], degree of breast cancer differentiation [73], age at breast cancer diagnosis [74], and receptor status of breast cancer tumors [75,76]. Likewise, *CYP19A1 *rs936306 may be associated with breast cancer disease-free survival [77]. Case-control studies of breast cancer risk related to *ESR1 *rs2234693 [73,76,78-87] and in relation to *CYP19A1 *rs936306 [77,88] are conflicting. Inconsistent results of breast cancer case-control studies are likely due to differences in ethnicity and menopausal status of participants across studies. Reasons exist to suspect that associations of SNPs with mammographic density may vary by BMI, as we found for *CYP1A1 *rs2606345. Sex steroid metabolism (for example, peripheral aromatization of androstenedione) varies by BMI, so that effects of sex steroid SNPs on breast tissue may be more pronounced among obese women. Although prior studies have not examined whether associations of *CYP1A1 *rs2606345 with mammographic density vary by BMI, a prior study reported that the association of an *ESR1 *SNP with increased breast cancer risk was apparent only among women with BMI greater than 25 kg/m^2 ^[69].

Strengths of our study included its multiethnic study population, use of validated and reproducible mammographic density-assessment techniques, rigorous attention to genotyping methods, and collection of detailed information regarding key covariates related to mammographic density. However, this study did not directly assess sex steroid activity in breast tissue samples. Furthermore, although our sample size was relatively large, its heterogeneity may have precluded detection of statistically significant race-specific associations or interactions of SNPs with mammographic density. Finally, the observational study design precluded coordination of mammographic density with menstrual-cycle phase. Relations between SNPs and mammographic density may have been diluted because we analyzed mammograms taken during varying menstrual phases. Breasts are more radiographically dense during the luteal phase [89-91], although a recent study found that variation in mammographic density over the menstrual cycle may be subtle (that is, may not be statistically significant) [92].

## Conclusions

In conclusion, SNPs involving sex steroid metabolism enzymes and ESR1 may be associated with mammographic density in pre- and early perimenopausal women. Future studies relating these SNPs to mammographic density not only should adjust for BMI but also should consider interactions by BMI. The mechanisms underlying the association (for example, increased proliferation of epithelial and stromal cells) require elucidation. Because these enzymes and ESR1 are expressed in target tissues, these SNPs (or genetic factors with which they are in linkage disequilibrium) may alter breast cancer risk by altering mammographic density. These findings inform the understanding of biologic influences on mammographic density, a strong risk factor for breast cancer.

## Abbreviations

BMI: body mass index; CYP: cytochrome P450; ESR: estrogen receptor; HSD: hydroxysteroid dehydrogenase; HWE: Hardy Weinberg equilibrium; SNP: single-nucleotide polymorphism; SWAN: Study of Women's Health Across the Nation.

## Competing interests

The authors declare that they have no competing interests.

## Authors' contributions

CC contributed to study conception, study design, analysis and interpretation of data, manuscript drafting, and revision. JS and MS contributed to study design. MS, SC, EG, LH, LB, MS, GG, and JS contributed to analysis and interpretation of data and manuscript revision.
